# Acute effects of single dose transcranial direct current stimulation on muscle strength: A systematic review and meta-analysis

**DOI:** 10.1371/journal.pone.0209513

**Published:** 2018-12-26

**Authors:** Eduardo Lattari, Bruno R. R. Oliveira, Renato Sobral Monteiro Júnior, Silvio Rodrigues Marques Neto, Aldair J. Oliveira, Geraldo A. Maranhão Neto, Sergio Machado, Henning Budde

**Affiliations:** 1 Physical Activity Sciences Post-Graduate Program (PGCAF), Salgado de Oliveira University (UNIVERSO), Niterói, RJ, Brazil; 2 Brazilian Institute of Medicine of Rehabilitation, Rio de Janeiro, RJ, Brazil; 3 Physical Education and Sport Department, State University of Montes Claros, Montes Claros, Minas Gerais, Brazil; 4 Post-graduation Program of Health Sciences, State University of Montes Claros, Montes Claros, Minas Gerais, Brazil; 5 School of Physical Activity, Rural Federal University of Rio de Janeiro, Rio de Janeiro, Brazil; 6 Faculty of Human Sciences, Medical School Hamburg, Hamburg, Germany; 7 Lithuanian Sports University, Kaunas, Lithuania; 8 Physical Activity, Physical Education, Health and Sport Research Centre (PAPESH), Sports Science Department, School of Science and Engineering, Reykjavik University, Reykjavik, Iceland; Nottingham Trent University, UNITED KINGDOM

## Abstract

Previous studies investigating the effects of transcranial direct current stimulation (tDCS) on muscle strength showed no consensus. Therefore, the purpose of this article was to systematically review the literature on the effects of single dose tDCS to improve muscle strength. A systematic literature search was conducted on PubMeb, ISI Web of Science, SciELO, and Scopus using search terms regarding tDCS and muscle strength. Studies were included in accordance with Population, Intervention, Comparison, Outcomes, and Setting (PICOS) including criteria. Healthy men and women, strength training practitioners or sedentary were selected. The acute effects of single dose anode stimulus of tDCS (a-tDCS) and the placebo stimulus of tDCS (sham) or no interventions were considered as an intervention and comparators, respectively. Measures related to muscle strength were analyzed. To conduct the analyses a weighted mean difference (WMD) and the standardized mean difference (SMD) were applied as appropriate. A total of 15 studies were included in this systematic review and 14 in meta-analysis. Regarding the maximal isometric voluntary contraction (MIVC), a small effect was seen between tDCS and Sham with significant difference between the conditions (SMD = 0.29; CI_95%_ = 0.05 to 0.54; Z = 2.36; p = 0.02). The muscular endurance measured by the seconds sustaining a percentage of MIVC demonstrated a large effect between tDCS and Sham (WMD = 43.66; CI_95%_ = 29.76 to 57.55; Z = 6.16; p < 0.001), showing an improvement in muscular endurance after exposure to tDCS. However, muscular endurance based on total work showed a trivial effect between tDCS and Sham with no significant difference (SMD = 0.22; CI_95%_ = -0.11 to 0.54; Z = 1.32, p = 0.19). This study suggests that the use of tDCS may promote increase in maximal voluntary contraction and muscular endurance through isometric contractions.

## Introduction

Muscle strength is underpinned by a combination of morphological and neural factors including motor unit recruitment, rate coding, motor unit synchronization, neuromuscular inhibition, muscle cross-sectional area, and musculotendinous stiffness [1]. Several evidences show the importance of muscular strength for health, considering that it may contribute in the improvement of different health factors such as a reduction in cardiovascular risk factors (triglycerides, LDL-cholesterol, glucose and blood pressure) [2, 3], as well as low muscular strength has been associated with increased mortality in adulthood [4]. Addition, muscular strength is one of the most important factors for physical performance in different sports [5]. Therefore, the maintenance and increase of muscular strength is recommended for athletes and non-athletes, being necessary a physical stimulus to obtain these objectives.

For decades, the literature has investigated different methods of training that optimize the increase in muscle strength in athletes and non-athletes [6–9]. Although different methods of training are relevant in increasing muscle strength, due to the increasing popularity of resistance training, a wide variety of ergogenic resources have been used for this purpose [10–12]. In this regard, the neuromodulation techniques also have been used as ergogenic aids with promising results in improving muscle strength compared to placebo stimulus (sham) [13–17].

The transcranial direct current stimulation (tDCS) consists of a noninvasive electrical stimulus that promotes changes in the resting potential of the neuronal membrane [18]. tDCS is non-invasive, well-tolerated [19] and produces acute changes in brain excitability by 10–30 minutes of tDCS at 1–2 mA and can last over an hour after a tDCS session [18, 20, 21]. This electrical stimulus can be applied on different areas of the cerebral cortex, having been investigated regarding its effects on muscle strength. Nonetheless, there is still no consensus in this matter [13, 15–17, 22, 23].

Previous studies have demonstrated that anodal tDCS (a-tDCS) was effective in promoting acute increases in submaximal strength (i.e.: muscular endurance) [14–16, 24]. Moreover, studies showed that a-tDCS was not capable of increasing the total work of knee extension and flexion in young healthy individuals [25] and muscular endurance with isometric muscle actions [23]. Concerning maximum strength, the results demonstrated greater pinch force in the toe [17], muscle power [13], and no change after the use of a-tDCS [16, 23].

These results suggest that a-tDCS could be useful as an auxiliary tool for muscle strength [26]. However, the effects of a-tDCS on different muscle groups and different types of muscle strength have shown contradictory results. Nonetheless, there are methodological differences regarding the stimulated area, current intensity and duration of a-tDCS [13–16, 23, 25]. Given the aforementioned information regarding the importance of muscle strength, identifying a safe ergogenic aid to optimize muscle strength is of extreme interest to athletes, coaches, researchers and may be an easy and helpful strategy for such [26]. In addition, non-athlete individuals with different fitness levels and health conditions may benefit from this method considering that the maintenance and improvement of muscle strength is desirable for different populations [24, 27]. Therefore, the purpose of this article was to systematically review the literature on the effects of single dose a-tDCS on improving muscle strength.

## Methods

The method of this study was designed and reported according to the recommendations of the Preferred Reporting Items for Systematic reviews and Meta-Analyses (PRISMA) [28] and the Cochrane Handbook for Systematic Reviews of Interventions [29].

### Protocol and registration

This study was not registered.

### Eligibility criteria

Studies were included in accordance with Participants, Intervention, Comparison, Outcomes, and Setting (PICOS) inclusion criteria:

1. Participants: Healthy men and women, strength training practitioners or sedentary, with no history of bone, muscle or joint injury and no psychiatric illness.
2. Intervention: Was utilized the acute effects of single dose the anode stimulus of tDCS (a-tDCS).
3. Comparators: The placebo stimulus of tDCS (sham) or no interventions were considered (control).
4. Outcomes: acute effects of measures related to muscle strength as the maximum muscle strength, muscular endurance, and muscle power were analyzed. Isometric and dynamic contractions were accepted.
5. Study Design: randomized and non-randomized trials, using either cross-over or parallel group designs, comparing an intervention encompassing a-tDCS with a sham group on muscle strength. Conference abstracts, dissertations, theses, book chapters, and articles published in non–peer-reviewed journals were not included.

Our analysis was confined to studies published in English and Portuguese languages, respectively.

### Information sources

A systematic literature search was conducted between June 20, 2018 and July 24, 2018. The following databases were used: PubMed, ISI Web of Science (Web of Science Core Collection), SciELO, and Scopus. No filters were applied in the search.

### Search strategy

Search terms were defined according to intervention (tDCS) and outcomes (muscle strength). The following search query was used on PubMed:

("transcranial direct current stimulation"[MeSH] OR transcranial direct current stimulation*[All Fields] OR "tDCS"[MeSH] OR "tDCS"[All Fields] OR Stimulation tDCS [MeSH] OR Stimulation tDCS*[All Fields] OR Transcranial Electrical Stimulation [MeSH] OR Transcranial Electrical Stimulation*[All Fields]) AND ("Muscle strength"[MeSH] OR Muscle strength*[All Fields]).

In the Web of Science and Scopus databases, the search was performed using the same terms combined in different searches as follows: a. transcranial direct current stimulation and muscle strength; b. tDCS and muscle strength; c. Stimulation tDCS and muscle strength; and d. transcranial electrical stimulation and muscle strength.

For the search using the SciELO, the same combined terms were translated to Portuguese through the Health Sciences Descriptors (DeCS). Included reports and important reviews regarding tDCS and muscle strength were manually screened for additional relevant studies. Experts on the field, including authors from the included reports, were also requested to suggest any additional trials in order to ensure that the review was as comprehensive and up-to-date as possible.

### Selection of studies

A spreadsheet was used to include the extracted data. After merging search results and discarding duplicates, two researchers (EL and BRRO) independently screened titles and abstracts in order to identify relevant studies. Full-text articles of the included reports were retrieved and independently assessed for eligibility by the two researchers according to the previously described criteria. A consensus meeting was performed in case of disagreement regarding any report and a third researcher (RSMJ) completed the decision when required. When it was not possible to retrieve full-text articles, authors were contacted using email and Research Gate in order to provide the required report. After three failed attempts to obtain response from the respective authors, the report was excluded from analysis.

### Data extraction

The following data was extracted from the articles: participant characteristics (sample size, gender, drop-outs, age, and previous experience with resistance training), tDCS intervention protocol (stimulated area, electrode size, current intensity, and session duration), resistance exercise characteristic (joint movement, type of contraction, and muscle strength test), and main outcomes. To minimize the risk of bias in data extraction, data was extracted twice by the same author.

### Assessment of risk of bias in included studies

Risk of bias was judged based on the criteria described on the *Cochrane Handbook for Systematic Reviews of Interventions*, version 5.1.0 [29]. The following criteria were evaluated:

- Selection bias: Random sequence generation (inadequate randomization procedures) and allocation concealment (inadequate concealment of allocations prior to assignment).
- Performance bias: blinding of participants and personnel (knowledge of the allocated interventions by participants and personnel).
- Detection bias: blinding of outcome assessments (knowledge of the allocated interventions by outcome evaluators).
- Attrition bias: incomplete outcome data (amount, nature or handling of incomplete data).
- Reporting bias: selective outcome reporting (differences between reported and unreported findings).
- Other bias: bias due to problems not covered elsewhere in the table (Low risk- The study appears to be free of other sources of bias; High risk- has been claimed to have been fraudulent or had a potential source of bias related to the specific study design used; Unclear risk- Insufficient rationale or evidence that an identified problem will introduce bias or Insufficient information to assess whether an important risk of bias exists).

Two researchers (EL and BRRO) independently assessed the included trials, rating each of the previously described factors with low, high, or unclear risk of bias according to the criteria defined by Higgins [29]. Again, a consensus meeting was performed in order to discuss rating disagreements and a third researcher (RSMJ) ensured the final decision when required.

### Meta-analyses

To conduct the analyses we extracted data related to strength (maximal isometric voluntary contraction–MIVC) and muscular endurance (time to exhaustion in seconds sustaining a percentage of MIVC–TTE-%MIVC, and total work–TW). MIVC was measured by authors with different units (e. g. N, N.m or N/Kg), TTE- % MIVC in seconds, and TW in volume-load and joules.

A weighted mean difference (WMD) and the standardized mean difference (SMD) were applied as appropriate. The heterogeneity index (I^2^) was checked to detect discrepancies among studies. A fixed or random order effect model was carried out according to Higgins [29]. All analyses were performed using Review Manager 5.3. In addition, Cohen’s Effect Size [30] was used to classify the results.

## Results

### Study selection

**Fig 1:**
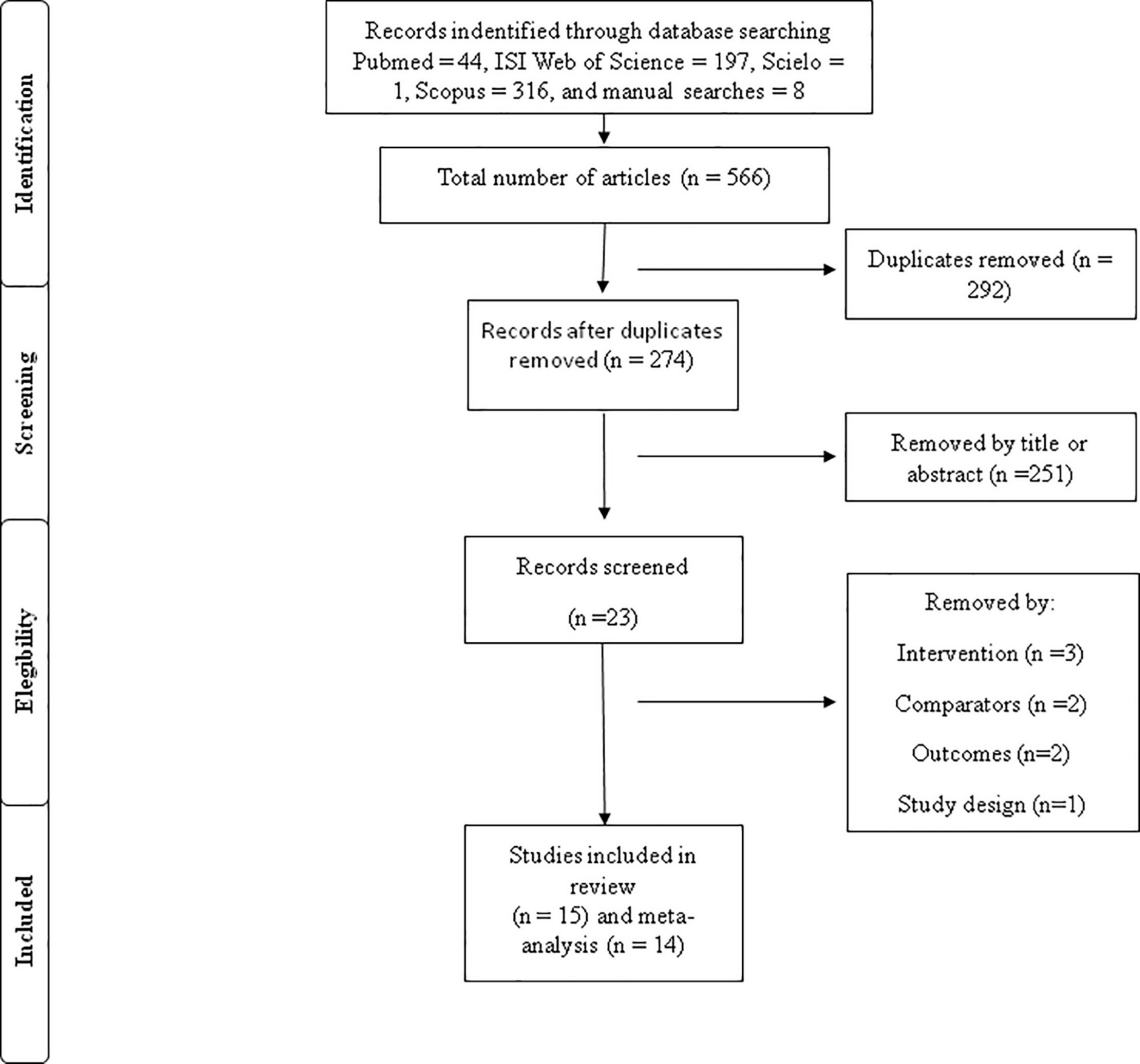
Flowchart of outcomes of search strategy.

The results identified a total of 566 articles (44 in the PubMed, 197 in the ISI Web of Science, one in the SciELO, 316 in the Scopus, and eight through manual searches). After the process of removal of duplicate articles (n = 292), a total of 274 articles remained. 251 articles were removed by title and / or abstract, remaining a total of 23 articles. Eligibility criteria determined the exclusion of seven articles [Intervention (n = 3); Comparators (n = 2); Outcomes (n = 2); Study Design (n = 1)]. After this removal process, 15 articles were included for systematic review and 14 for meta-analysis. The study conducted by Lattari et al. [13] was removed from the meta-analysis as the only article to investigate the acute effects of single dose a-tDCS on muscle power. Study selection flow chart is presented in Fig 1.

### Study characteristics

#### Participant characteristics

Characteristics of the participants in the included studies are described in Table 1. There’s a total of 219 subjects that participated in the acute tDCS studies on muscle strength. Regarding tDCS conditions, a-tDCS conditions had sample sizes between 8 and 22 [26, 31], with a total of 204 subjects among studies. Control conditions had sample sizes between 8 and 22 [26, 31], with a total of 210 subjects among studies. There were a total of 2 dropouts (20%) in the Tanaka’s study [17] and 1 dropouts (4.5%) in the Radel’s study [31]. Regarding gender, most studies had more male participants than women (as expected). In most a-tDCS and control conditions, subjects’ average age was between 16±0.9 [32] and 27.7±8.4 years old [23]. It is also important to note that only five studies [13–15, 25, 26] reported duration in experience strength training, a factor that may clearly play a role in a-tDCS response.

**Table 1:** Participant characteristics.

Reference	N	Drop-outs (N;%)	Gender	Age	Experience with ST
Cogiamanian et al.(2007)	a-tDCS = 9 control = 15	None	a-tDCS = 5 (F) and 4 (M) control = 9 (F) and 6 (M)	24.3	None of the subjects were engaged in competitive sport activities specifically involving elbow flexor muscles
Kan et al. (2013)	a-tDCS = 15 sham = 15	None	a-tDCS = 15 (M) sham = 15 (M)	27.7 (±8.4)	Not reported
Abdelmoula et al. (2016)	a-tDCS = 11 sham = 11	None	a-tDCS = 8 (M) and 3 (F) sham = 8 (M) and 3 (F)	25.0 (±1.8)	None of the subjects were engaged in regular strength training programs
Radel et al. (2017)	a-tDCS = 22 sham = 22	1 (4.5%)	a-tDCS = 13 (M) and 9 (F) sham = 13 (M) and 9 (F)	21.30 (±0.4)	Not reported
Flood et al. (2017)	a-tDCS = 12 sham = 12	None	a-tDCS = 8 (M) sham = 8 (M)	24.4 (±3.8)	Recreationally active and not engaged in regular strength training programs
Hazime et al.(2017)	a-tDCS = 8 sham = 8	None	a-tDCS = 8 (F) sham = 8 (F)	19.7 (±2.3)	Handball athletes (31 weeks of ST)
Vargas et al. (2017)	a-tDCS = 20 sham = 20	None	a-tDCS = 20 (F) sham = 20 (F)	16.1 (±0.9)	> five years of training in soccer (not reported with ST)
Angius et al.(2016)	a-tDCS = 9 sham = 9	None	a-tDCS = 9 (M) sham = 9 (M)	23.0 (±2.0)	Recreationally active (not reported with ST)
Tanaka et al. (2009)	a-tDCS = 10 sham = 10	2 (20%)	a-tDCS = 8 (M) and 2 (F) sham = 8 (M) and 2 (F)	23.8 (20–35)	Not reported
Lattari et al. (2016)	a-tDCS = 10 sham = 10	None	a-tDCS = 10 (M) sham = 10 (M)	26.5 (±5.0)	> six months
Lattari et al. (2017)	a-tDCS = 10 sham = 10	None	a-tDCS = 10 (M) sham = 10 (M)	22.1 (±3.8)	47.8±22.7 months
Lattari et al. (2018)	a-tDCS = 15 sham = 15	None	a-tDCS = 15 (F) sham = 15 (F)	24.5 (±3.3)	> one year
Montenegro et al. (2015)	a-tDCS = 14 sham = 14	None	a-tDCS = 14 (M) sham = 14 (M)	26.0 (±4.0)	> six months
Sales et al. (2016)	a-tDCS = 19 sham = 19	None	a-tDCS = 19 (M) sham = 19 (M)	25.1 (±3.9)	Physically active (not reported with ST)
Ciccone et al. (2018)	a-tDCS = 20 sham = 20	None	a-tDCS = 10 (M) and 10 (F) sham = 10 (M) and 10 (F)	21.0 (±1.5)	Recreationally active (not reported with ST)

N- number of participants; M- male; F- female; %- percentage; ST- Strength training; >- greater.

### Intervention protocols and control condition

The characteristics of the included a-tDCS protocols and respective control conditions are described in Table 2. The a-tDCS intervention protocol presented stimulation of motor cortex (MC) [13, 16, 17, 23–26, 32–34], dorsolateral prefrontal cortex (DLPFC) [14, 15, 31], and temporal cortex (TC) [35, 36]. Two studies used high-definition tDCS for electrodes montage [31, 34]. The positioning of the electrodes were placed in a 4 X 1 ring configuration with the centre electrode positioned over the hand cerebral cortex (anodal) and return electrodes positioned in a ring around the centre anode (cathodal) at a radius of approximately 5 cm and 4 cm [31, 34]. Electrodes with different sizes were used in the stimulated area, between 12 and 35 cm^2^. The electrodes size in a 4 X 1 ring configuration was reported with approximate diameter of 1.1 cm [31, 34]. Two studies used an electric current intensity of 1.5 mA [16, 33] and all others applied a current intensity of 2 mA [13–15, 17, 23–26, 31, 32, 34–36]. Furthermore, session duration was 10 [16, 17, 23, 24, 31, 33] to 20 minutes [13–15, 25, 26, 32, 34–37].

**Table 2:** Study protocols.

Reference	Intervention protocol (a-tDCS)	Control	Resistance exercise characteristic	Main outcomes
	Stimulatory electrode and reference; Electrode size (cm^2^); Current intensity (mA); Duration (min)	Duration (s)	Joint movement; Type of contraction; Muscle strength test	
Cogiamanian et al., (2007)	Right MC (stimulus) and right shoulder (reference); 35 cm^2^; 1.5 mA; 10 min	CG	Left elbow flexors; Isometric; MIVC (N) and TTE with 35% of the MIVC (s)	MIVC: ≠ between the conditions TTE: a-tDCS > CG (p<0.05)
Kan et al., (2013)	Right MC (stimulus) and right shoulder (reference); 24 cm^2^; 2.0 mA; 10 min	30 (s) (sham)	Left elbow flexors; Isometric; MIVC (N.m) and TTE with 30% of the MIVC (s)	MIVC: ≠ between the conditions TTE: ≠ between the conditions
Abdelmoula et al. (2016)	Left MC (stimulus) and right shoulder (reference); 35 cm^2^; 1.5 mA; 10 min	90 (s) (sham)	Right elbow flexors; Isometric; MIVC (N) and TTE with 35% of the MIVC (s)	MIVC: ≠ between the conditions TTE: a-tDCS > sham (p<0.05)
Radel et al. (2017)	Two Positioning of the electrodes (4x1): First- Right MC (stimulus) and four cathodal electrodes placed at a distance of 4 cm around the anode (reference); Second- Right DLPFC (stimulus) and four cathodal electrodes placed at a distance of 4 cm around the anode (reference); radius ≈ 1.1 cm; 2.0 mA; 10 min	30 (s) (sham)	Left elbow flexors; Isometric TTE with 35% of the MIVC (s)	TTE: ≠ between the conditions
Flood et al. (2017)	Positioning of the electrodes (4x1) MC contralateral to the non-dominant side (stimulus, C3 or C4) and four cathodal electrodes placed at a distance of 5 cm around the anode (reference); radius ≈ 1.1 cm; 2.0 mA; 20 min	At the start and at the end (2 mA in ramping)	Non-dominant knee extensors; Isometric; MIVC (N.m) and TTE with 30% of the MIVC (s)	MIVC: ≠ between the conditions TTE: ≠ between the conditions
Hazime et al., (2017)	MC dominant limb (stimulus) and ipsilateral OBF (reference); 35 cm^2^; 2.0 mA; 20 min	30 (s) (sham)	Internal and external rotator; Isometric; MIVC (N/kg)	MIVC (internal and external rotator): a-tDCS > sham (p<0.05)
Vargas et al., (2017)	Lef and right MC (stimulus) and ipsilateral OBF (reference); 35 cm^2^; 2.0 mA; 20 min	30 (s) (sham)	Knee extensors; Isometric; MIVC (N/kg) in dominant and non-dominant limb	Dominant a-tDCS > sham (p<0.05) Non-dominant ≠ between the conditions
Angius et al., (2016)	Two Positioning of the electrodes: First- Left MC (stimulus) and right OBF (reference); Second- Left MC (stimulus) and left shoulder (reference); 12 cm^2^; 2.0 mA; 10 min	30 (s) (sham)	Right knee extensors; Isometric; MIVC (N.m) and TTE with 20% of the MIVC (s)	MIVC: NR TTE: Second position- a-tDCS > sham (p<0.05); First position ≠ between the conditions
Tanaka et al., (2009)	Right MC (stimulus) and right OBF (reference); 35 cm^2^; 2.0 mA; 10 min	30 (s) (sham)	Adduction between the left great toe and the digitus secundus (leg pinch force) Adduction between the index finger and the thumb pad of the left hand (hand pinch force); Isometric; PF (N)	PF (Leg): a-tDCS > sham (p<0.01) PF (Hand): ≠ between the conditions
Lattari et al., (2016)	Left DLPFC (stimulus) and right OBF (reference); 35 cm^2^; 2.0 mA; 20 min	30 (s) (sham)	elbow flexors; Dynamic; Volume-load (kg)	*a-tDCS > sham (p<0.05)
Lattari et al., (2017)	Central MC (stimulus) and right OBF (reference); 35 cm^2^; 2.0 mA; 20 min	30 (s) (sham)	Ankle, hip, and knee extensors; Dynamic; Muscle power (W)	≠ between the conditions
Lattari et al., (2018)	DLPFC (stimulus) and right OBF (reference); 35 cm^2^; 2.0 mA; 20 min	30 (s) (sham)	Ankle, hip, and knee extensors; Dynamic; Volume-load (kg)	a-tDCS > sham (p<0.05)
Montenegro et al., (2015)	Left MC (stimulus) and right OBF (reference); 35 cm^2^; 2.0 mA; 20 min	30 (s) (sham)	Knee extensors and flexors; Dynamic; Isokinetic testing (angular velocity of 60°∙s^-1^: Total work (J) and peak torque (N.m)	*Total work: ≠ between the conditions *Peak torque: ≠ between the conditions
Sales et al., (2016)	Left TC (stimulus) and right OBF (reference); 35 cm^2^; 2.0 mA; 20 min	30 (s) (sham)	knee extensors; Dynamic; Isokinetic testing (angular velocity of 180°∙s^-1^ and 60°∙s^-1^): Total work (J) and peak torque (N.m)	*Total work: a-tDCS > sham (p<0.05) *Peak torque: ≠ between the conditions
Ciccone et al., (2018)	Two Positioning of the electrodes: First- Left TC (stimulus) and right OBF (reference); Second- Right TC (stimulus) and left OBF (reference); 25 cm^2^; 2.0 mA; 20 min	30 (s) (sham)	knee extensors; Dynamic; Isokinetic testing (angular velocity of 180°∙s^-1^): Average work (Nm.s)	≠ between the conditions

a-tDCS- anodal transcranial direct current stimulation; CG- control group; cm^2^- square centimeter; NR- mA- milliamps; min- minutes; s- seconds; MC- motor cortex; DLPFC- dorsolateral prefrontal cortex; OBF- orbitofrontal cortex; ≈ approximately; MIVC- Maximal Isometric Voluntary Contraction; PF- Pinch Force; N- Newtons; N.m- Newtons per meter; N/kg- Newtons per kilogram (normalized by the body mass of each participant); Kg- Kilogram; J- Joules; %- percentage; Nm.s- Newtons meter per second; TTE = time to exhaustion; NR- not reported

In the control conditions, only one study used no placebo stimulus (sham) [16] and all the others utilized the sham condition [13–15, 17, 23–26, 31–36]. Twelve studies used as a sham stimulus a duration of 30 seconds [13–15, 17, 23–26, 31, 32, 35, 36]. The positioning of the electrodes was equal of a-tDCS condition.

### Resistance exercise characteristic

The resistance exercise characteristic showed that isometric [16, 17, 23, 24, 26, 31–34] and dynamic contractions [13–15, 25, 35, 36] were used. For dynamic exercises were used isokinetic testing [25, 35, 36], muscle action against a constant load [14, 15], and muscle power. The following joint movements were used: elbow flexors [14, 16, 23, 31, 33], internal and external rotator [26], knee extensors [24, 32, 34–36], adduction between the left great toe and the digitus secundus and adduction between the index finger and the thumb pad of the left hand [17], extension of the ankle, hip, and knee [13, 15], knee extensors and flexors [25]. The changes in muscle strength were investigated through tests of muscular endurance [14–16, 23–25, 31, 33–36], and maximum strength [16, 17, 23, 24, 26, 32–34]. Only one study investigated the effects of single dose tDCS on the muscle power [13].

### Results of individual studies

The main outcome, presented in four studies, was that no difference was observed between the a-tDCS and sham conditions in maximal isometric voluntary contraction (MIVC) tests [16, 23, 33, 34]. One study showed that MIVC of the internal and external rotator was greater in the a-tDCS condition compared to sham condition [26]. In another study, it was also possible to observe that MIVC of the dominant knee extensor was greater in the a-tDCS condition compared to sham condition [32]. However, no difference was observed between the a-tDCS and sham conditions for non-dominant knee extensor [32]. The leg pinch force (PF) was greater during the a-tDCS application, when compared to a sham condition [17]. In two studies, it was not possible to affirm that significant differences between the a-tDCS and sham conditions occurred, because the data related to hand pinch force [17] and knee extension [24] were not reported.

Regarding muscular endurance, a-tDCS was greater than the sham conditions in six studies [14–16, 24, 33, 35]. These differences were observed in isometric contraction [16, 24, 33], muscle action against a constant load [14, 15] and isokinetic [35] strength tests. In six studies no difference were observed between conditions for muscle endurance through isometric contraction [23, 24, 31, 34], and isokinetic [25, 36] strength tests. For muscle power, one study showed that there was no significant difference between conditions [15].

### Risk of bias within and across studies

Risk of bias assessment for each included trial is presented on Table 3. Only two trials provided no sufficient information about the way the allocation sequence was generated [14, 16]. For allocation concealment procedures and blinding of outcome assessment, all trials were classified as low risk of bias. One study presented high risk of bias because the researchers were not blinded to experimental conditions [36]. Only three studies presented high risk of incomplete outcome data [17, 31, 35]. In Tanaka’s study [17], three subjects did not perform the hand pinch force task. One extreme value of endurance time was excluded by Radel's study [31]. In the research conducted by Sales et al [35] the data was reported only in figure and included in the discussion. Four studies showed high risk for selective reporting [14, 17, 24, 25]. The data of MIVC [17, 24], volume-load [14], total work, and peak torque [25] were not reported, but some were sent [14, 25]. Finally, one study was also classified with unclear risk of other bias as they did not describe if participants included in the trial had experience with strength training (ST), and also drop-out rates (20%) were quite substantial in the hand PF task [17].

**Table 3:** Risk of bias assessment.

Reference	Random Sequence Generation	Allocation Concealment	Blinding of Participants and Personnel	Blinding of Outcome Assessment	Incomplete Outcome Data	Selective Reporting	Other Bias
Cogiamanian et al.(2007)	Unclear	Low	Low	Low	Low	Low	Low
Kan et al.(2013)	Low	Low	Low	Low	Low	Low	Low
Abdelmoula et al. (2016)	Low	Low	Low	Low	Low	Low	Low
Hazime et al.(2017)	Low	Low	Low	Low	Low	Low	Low
Radel et al. (2017)	Low	Low	Low	Low	High (One subject not performed TTE task)	Low	Low
Flood et al. (2017)	Low	Low	Low	Low	Low	Low	Low
Vargas et al. (2017)	Low	Low	Low	Low	Low	Low	Low
Angius et al.(2016)	Low	Low	Low	Low	Low	High (MIVC not reported)	Low
Tanaka et al.(2009)	Low	Low	Low	Low	High (Two subjects not performed the hand PF task)	High (MIVC not reported)	Unclear
Lattari et al.(2016)	Unclear	Low	Low	Low	Low	High (volume-load not reported)	Low
Lattari et al.(2017)	Low	Low	Low	Low	Low	Low	Low
Lattari et al.(2018)	Low	Low	Low	Low	Low	Low	Low
Montenegro et al.(2015)	Low	Low	Low	Low	Low	High (total work and peak torque not reported)	Low
Sales et al. (2016)	Low	Low	Low	Low	High (Data reported only in figure and discussion)	Low	Low
Ciccone et al. (2018)	Low	Low	High (The researchers were not blinded to experimental conditions)	Low	Low	Low	Low

### Synthesis of results

#### MIVC

**Fig 2:**
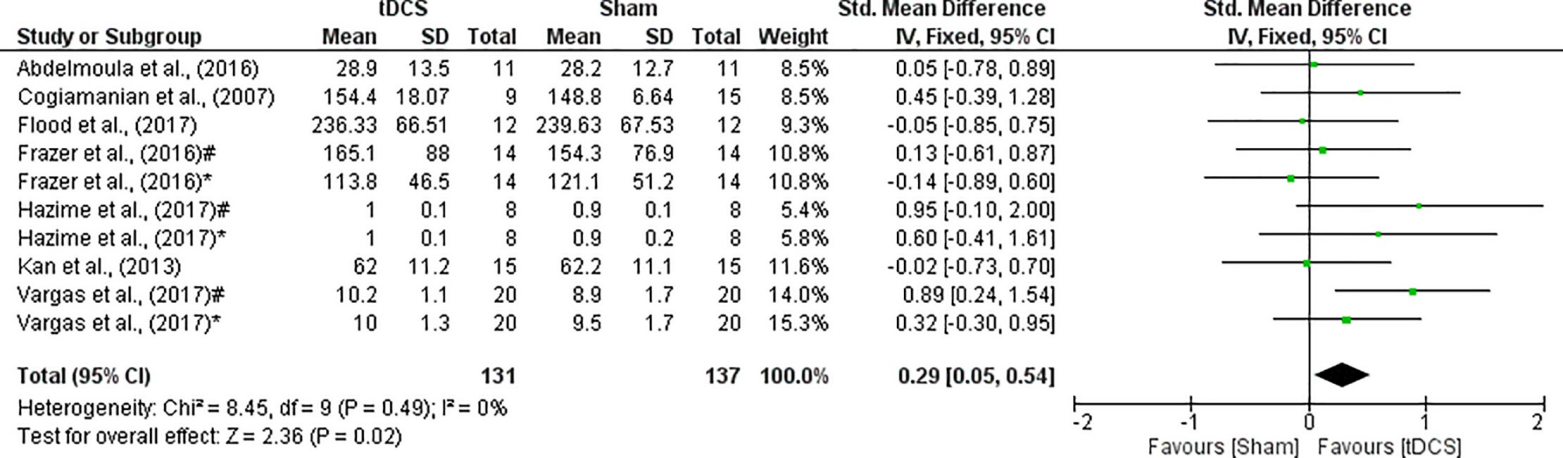
Forest plot showing a comparison of MIVC between tDCS and Sham. Hazime et al. (2017)^#^- internal rotador shoulder; Hazime et al. (2017)*- external rotador shoulder; Vargas et al. (2017)^#^- knee extensors dominant limb; Vargas et al. (2017)*- knee extensors non-dominant limb.

A SMD was performed to analyze MIVC due to the different measurements. The heterogeneity of this data was not significant (I^2^ = 0%; p = 0.49). For this reason, a fixed effect model was applied. A small effect was seen between a-tDCS and Sham on MIVC (SMD = 0.29; CI_95%_ = 0.05 to 0.54; Z = 2.36; p = 0.02) with significant difference between the conditions. All details of each study and the overall effect are shown in Fig 2.

#### TTE-%MIVC

**Fig 3:**
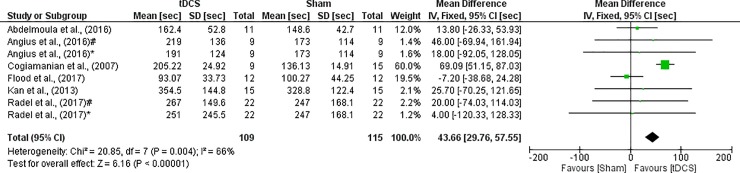
Forest plot showing a comparison of TTE-%MIVC between tDCS and Sham. Angius et al. (2016)^#^- electrode montages (shoulder); Angius et al. (2016)*- electrode montages (head); Radel et al. (2017)#—electrode montages (stimulus in right MC); Radel et al. (2017)*- electrode montages (stimulus in right DLPFC).

Muscular endurance was based on the seconds sustaining a percentage of MIVC. Hence, a WMD was used to analyze data. There was significant heterogeneity (I^2^ = 66%, p = 0.004), thus a fixed effect was applied. A large effect was seen between a-tDCS and Sham (WMD = 43.66; CI_95%_ = 29.76 to 57.55; Z = 6.16; p < 0.001), showing an improvement on muscular endurance in individuals who were submitted to a-tDCS (Fig 3).

#### TW

**Fig 4:**
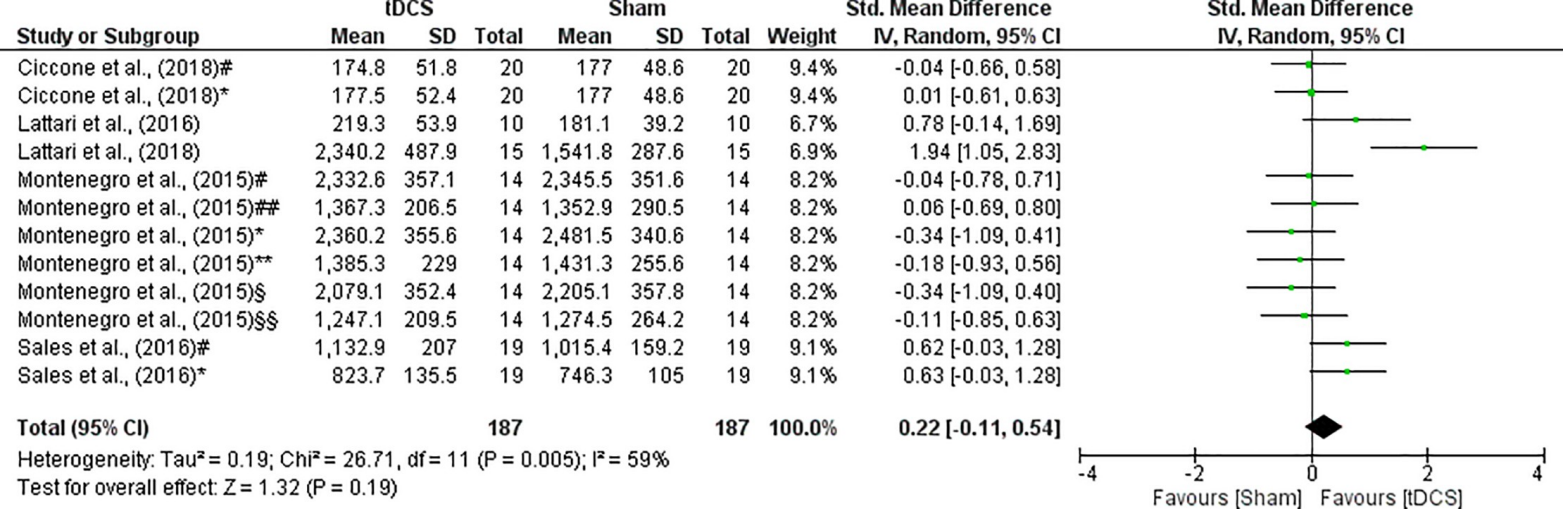
Forest plot showing a comparison of TW between tDCS and Sham. Ciccone et al. (2018)^#^- isokinetic muscle actions of the knee extensors in angular velocity of 180°.s^-1^ with stimulus applied on the left temporal cortex; Ciccone et al. (2018)*- isokinetic muscle actions of the knee extensors in angular velocity of 180°.s^-1^ with stimulus applied on the right temporal cortex; Montenegro et al. (2015)*- 1^st^ set of knee extensors; Montenegro et al. (2015)^§§^- 3^rd^ set of knee flexors; Montenegro et al. (2015)**- 1^st^ set of knee flexors; Montenegro et al. (2015)^##^- 2^nd^ set of knee flexors; Montenegro et al. (2015)^§^- 3^rd^ set of knee extensors; Montenegro et al. (2015)^#^- 2^nd^ set of knee extensors; Sales et al. (2016)^#^- isokinetic muscle actions of the knee extensors in angular velocity of 60°.s^-1^; Sales et al. (2016)*- isokinetic muscle actions of the knee extensors in angular velocity of 180°.s^-1^.

The authors of included studies showed data of TW in different units. A effect size showed a trivial effect (SMD = 0.22; CI_95%_ = -0.11 to 0.54) between a-tDCS and Sham with no significant difference (Z = 1.32, p = 0.19). The heterogeneity was significant (I^2^ = 59%, p = 0.005), hence a random effect model was applied. These results are shown in Fig 4.

## Discussion

The purpose of this article was to systematically review the effect of a-tDCS on muscle strength. The results showed that maximal voluntary contraction and muscular endurance through isometric contractions were improved with use of the a-tDCS. Regarding the muscular endurance through the total work, no change occurred as a consequence of a-tDCS. Our discussion was divided into three topics regarding study outcomes, MIVC, and endurance muscular (TTE-%MIVC, and total work).

### MIVC

In our meta-analysis it was possible to demonstrate a small effect for MIVC between tDCS and Sham (ES = 0.29). Results of individual studies showed that a-tDCS promoted greater MIVC of internal and external rotators [26], knee extensors (dominant limb) [32], and leg PF [17] during its application compared to sham condition. These studies stimulated the same area of the cortex (MC), current intensity (2 mA), and electrode size (35 cm^2^). The duration of the stimulus was different, Tanaka et al.[17] stimulated the subjects for 10 minutes while Vargas et al.[32] and Hazime et al. [26] stimulated for 20 minutes. One explanation could be that the increases of MIVC in healthy subjects indicate that a-tDCS may temporarily improve muscle strength beyond normal levels [17], even in athletes [26, 32]. We speculate that improvements in MIVC may be attributable to three main factors: increased cortical excitability, an increase in cross-activation and a decrease in short-interval intracortical inhibition (SICI) due to the a-tDCS [18, 38]. For example, when fifty-three healthy subjects were submitted to a-tDCS condition with 10 min and 2mA of current intensity, the results showed that a-tDCS facilitated MEPs whereas there was no significant effect of cathodal tDCS [39]. In fact, the addition of a-tDCS during unilateral strength training (ST + a-tDCS) was accompanied by significant increases in corticomotoneuronal excitability, decreases in SICI, and strength increase significantly greater than the ST + sham group [40]. On the other hand, the effects of a-tDCS on cortical excitability demonstrated large interindividual variability [39, 41]. Thereby, the plausible neurophysiological mechanism explaining the improvement on MIVC is not clear. Reduced pain, motivation, changes in muscle synergy, or modulatory effects on motor/premotor excitability pain, changes in muscle synergy, or modulatory effects on motor/premotor excitability are also speculated as possible factors [16].

Previous studies using MIVC measures showed no difference between the a-tDCS and sham conditions [16, 17, 23, 26, 32–34, 42]. The potential ergogenic effects of motor cortex targeted a-tDCS have been attributed to increased corticomotoneuronal excitability in the exercising limb [34]. However, the excitability after-effects do not linearly correlate with stimulation intensity, as lower intensities (0.5 and 1 mA) display equal or greater effects in comparison to higher intensities [41]. In addition, the results demonstrated in previous researches have suggested that MIVC improvement may not occur due to a ceiling effect on the capacity to produce force [16, 23].

### TTE-% MIVC

Regarding muscular endurance, through the meta-analysis it was possible to demonstrate that the tDCS generated improvements in muscular endurance in isometric contractions compared to the control condition, with a large effect size. It is important to note that the results presented high heterogeneity (I^2^ = 66%) and may represent substantial heterogeneity [43]. These results could possibly be occasioned by two main reasons. The first one is that the study of Cogiamanian et al.[16] presented a high weight of 60% and the second one is that few studies were included in the meta-analysis [16, 23, 24, 31, 33, 34]. Considering this information, these results should be interpreted with caution.

Three studies corroborated the positive effect of a-tDCS on muscular endurance [16, 24, 33]. Cogiamanian et al. [16] investigating the effect of tDCS compared to a control condition on elbow flexor isometric time to exhaustion (TTE) tasks. The participants received anodal tDCS and the control group did not receive any tDCS administration (no stimulation). Endurance time decreased significantly less after anodal than after no stimulation. Similarly, Angius et al. [24] compared the effect of two tDCS montages (head and shoulder) on TTE of knee extensors. In the head montage, anodal electrode was placed over the left motor cortex and the cathodal on contralateral forehead, while for the other montage, the anodal electrode was placed over the left motor cortex and cathodal electrode above the shoulder. tDCS was delivered for 10 min at 2.0 mA, after which participants performed an TTE test of the right knee extensors. TTE was significantly longer when a shoulder montage was used. Abdelmoula et al. [33] showed that TTE test with 35% of MIVC was significantly greater after a-tDCS than sham stimulation These variations in exercise performance arising from tDCS can be a consequence of different montages [16, 24, 33]. This is because the tDCS cathode decreases excitability over the area that it is placed [20]. Therefore, the cathodal electrode (i.e.reference) placed over the contralateral prefrontal area, rather than the opposite shoulder may have negated the positive effects of the anodal stimulation [16, 24, 33].

Results of individual studies showed no effect in four studies of a-tDCS on muscular endurance [23, 24, 31, 34]. These studies investigated the effects of tDCS on muscular endurance in the TTE task used low MIVC [16, 24, 33]. We theorize that improvements in muscular endurance in isometric contractions may be due to the low load used (35% and 20% of MIVC) because these results occurred in the absence of any change in neuromuscular or corticospinal parameters [16, 24, 33]. For example, eleven adults participated of submaximal voluntary contractions (35% maximal torque) performed to failure, with the right elbow flexor muscles [33]. The results show that the rates of increase in EMG, of both biceps brachii and brachioradialis muscles, were not influenced by stimulation conditions. Furthermore, the EMG of triceps brachii was also not influenced by stimulation conditions. In addition, a-tDCS increased the magnitude of biceps brachii activation at 37.5% and 50% of maximum [44]. However, anodal tDCS did not affect the voluntary EMG/force relationship of biceps brachii at 12.5% and 25% of MIVC. Nevertheless, these results are limited in terms of practical applications.

### TW

The muscular endurance measured by total work showed no significant difference between a-tDCS and Sham, and a trivial effect between the conditions. Of particular interest, in terms of the practical applicability [14, 15], the studies used strength exercises commonly performed in gym centers. Nevertheless, other studies involved isokinetic muscle actions [25, 35, 36] which are not so common in gym centers.

Two articles showed that tDCS generated improvements in total work with elbow flexor [14] and leg-press exercise [15]. It was also demonstrated that a-tDCS generated improvements in muscular endurance involving isokinetic muscle actions with knee extensors [25, 35, 36]. However, in two studies it was not possible to observe improvements in muscular endurance in isokinetic muscular actions after the use of a-tDCS [25, 36].

It is important to note the difference in the area stimulated between this studies [14, 15, 25, 35, 36]. In this regard, it was shown that the DLPFC can assist in sustained contractions when a failure was generated in output from the motor cortex [14, 27]. To maintain the required force, the input to the spinal motoneurons must be increased [45, 46] and during sustained submaximal contraction, the excitability of spinal motoneurons and the contractile capacity of the muscle fibers are reduced [47]. This failure to generate output from the motor cortex has been defined as supraspinal fatigue [48, 49]. In regarding to temporal cortex, the neurophysiological mechanism explaining the improvement on muscular endurance is not clear.

Besides, it has been shown that tDCS (2mA; 20min; MC) improved muscle power in strength trained individuals [13]. There were improvements in height, flight time, and peak power in the countermovement jump [13].

#### Limitations

The present study has several limitations and factors that may have influenced the results. (1) Limited number of studies included in review (n = 15) and meta-analysis (n = 14); (2) high heterogeneity presented in muscular endurance; and (3) differences regarding the strength task performed in dynamic contractions and stimulated cortical area.

The spatial specificity of the electrode montage applied via conventional tDCS limit the potential for comparisons to be made between the current findings. In this review, only two studies used high-definition tDCS for electrodes montage [31, 34]. The use of non-focal tDCS may influence other cortical areas, which could be responsible for the observed difference in muscle strength [33]. In fact, the reduction in muscle strength may arise because the central nervous system fails to drive the motoneurons adequately [46, 48]

In regarding to stimulated area, a number of studies investigated the role of other cortical regions in the regulation of muscle strength [15, 16, 23, 31, 36]. For example, the motor cortex is responsible for the output neural drive to the muscle [46]. Previous studies demonstrated that a-tDCS applied over the scalp of motor cortex resulted in an increase in the MIVC. However, despite the subject's maximal effort, motor cortical output at the moment is not sufficient to drive the motoneurons to produce maximal force from the muscle [46, 48–50]. Furthermore, the prefrontal cortex (PFC) is particularly active during a sustained contraction task [51]. In agreement with this suggestion, some researchers showed an increase in the muscle endurance after a-tDCS [14, 15]. On the other hand, no effects of the stimulation were observed on endurance time in elbow flexors [31]. Thereby, the cortical area stimulated presents important limitations.

Furthermore, the resistance exercise characteristic was different between studies. Abdelmoula et al. [33] suggests that a-tDCS does not act similarly on the mechanisms involved in the loss of MIVC and of sustained submaximal contraction. Agreeing to this suggestion, Enoka et al.[52] reported that the decline in MIVC does not directly explain the time to failure of a submaximal contraction. Our understanding of the interactions between the nervous system and muscle remains rather rudimentary. Among several limiting factors, individual variability in cortical excitability has received great attention in research [41]. In addition, a decrease in SICI also has received great attention [40].

Other important limitations of the study consist of the age, samples (males and females), psychological state, genetics, and time of day. Eleven very old individuals performed 3 maximal isometric elbow flexion contractions before and after 20 minutes of sham or a-tDCS [53]. The results showed that a-tDCS did not alter muscle strength in comparison to sham stimulation. The effect of a-tDCS in the very old is a question that is still to be addressed. Anodal stimulation to the DLPFC increased accuracy on the emotional perception test in females only [54] and psychological state an important role in training, competition, tolerance of pain and motivation [55]. In general, genetic diversity is a decisive biological basis of variations in neuronal network functioning after tDCS. The functional Val(108/158)Met polymorphism in the COMT gene, demonstrated to specifically predict the effect of tDCS on cognitive control [56]. The time of day in which the experimental conditions are performed can also influence the effects of tDCS. Anodal tDCS compared to sham stimulation improved recollection accuracy in the morning [57]. Future studies should consider these factors when investigating the effects of tDCS on muscle strength.

## Conclusions

This study suggests that the use of a-tDCS may increase the maximal voluntary contraction and muscular endurance through isometric contractions in novice and advanced strength training. It can be used as an ergogenic aid by coach and personal trainers especially in tasks involving isometric contractions. Thereby, a-tDCS could be applied as a complementary tool in muscle strengthening programs.

## Supporting information

S1 Checklist PRISMA checklist. (DOCX)
