# Correction: Acute effects of single dose transcranial direct current stimulation on muscle strength: A systematic review and meta-analysis

**DOI:** 10.1371/journal.pone.0229858

**Published:** 2020-03-02

**Authors:** Eduardo Lattari, Bruno R. R. Oliveira, Renato Sobral Monteiro Júnior, Silvio Rodrigues Marques Neto, Aldair J. Oliveira, Geraldo A. Maranhão Neto, Sergio Machado, Henning Budde

There are errors in the reporting of the studies included in this systematic review and meta-analysis. The authors provide the following clarification:

The potential error of our article was to include data from the study conducted by Frazer et al. (2016) in the Results section regarding the maximal isometric voluntary contraction (MIVC). Eligibility criteria determined the exclusion of this article by the intervention (n = 3). In the study by Frazer et al. (2016), 4 consecutive sessions of anodal and sham transcranial direct current stimulation (tDCS) were used. In fact, the objective of our study was systematically to review the literature on the effects of single dose tDCS to improve muscle strength. Based on this objective, we performed the data analysis related to the MIVC. The new results of the MIVC showed that the heterogeneity of the data were not significant (I^2^ = 0%; p = 0.48), and there was a significant difference between a-tDCS and sham-tDCS with a small effect size (SMD = 0.38; CI_95%_ = 0.10 to 0.65; Z = 2.68; p = 0.007). Exclusion of the study by Frazer et al. (2016) was not statistically significant compared to previous results (SMD = 0.29; CI_95%_ = 0.05 to 0.54; Z = 2.36; p = 0.02).Thus, the results of this study remain the same.

The authors provide updates to sentences in the Abstract, Results, and Discussions sections to correct these errors. Please see the location of the error, the original text, and the author-corrected text here.

**Table:** 

Location	Original text	Corrected text
Abstract, ninth and tenth sentences	“A total of 15 studies were included in this systematic review and 14 in meta-analysis. Regarding the maximal isometric voluntary contraction (MIVC), a small effect was seen between tDCS and Sham with significant difference between the conditions (SMD = 0.29; CI_95%_ = 0.05 to 0.54; Z = 2.36; p = 0.02).”	“A total of 15 studies were included in this systematic review and 13 in meta-analysis. Regarding the maximal isometric voluntary contraction (MIVC), a small effect was seen between tDCS and Sham with significant difference between the conditions (SMD = 0.38; CI_95%_ = 0.10 to 0.65; Z = 2.68; p = 0.007).”
Results, Study selection subsection, fifth sentence	“After this removal process, 15 articles were included for systematic review and 14 for meta-analysis.”	“After the removal process, 15 articles were included for systematic review and 13 for meta-analysis.”
Results, Study selection subsection, sixth sentence	“The study conducted by Lattari et al. [13] was removed from the meta-analysis as the only article to investigate the acute effects of single dose a-tDCS on muscle power.”	“The study conducted by Lattari et al. [13] was removed from the meta-analysis as the only article to investigate the acute effects of single dose a-tDCS on muscle power. Tanaka and collaborators [17] search has been removed because the data of MIVC not reported.”
Results, Synthesis of results subsection, MIVC subheading, second sentence	“The heterogeneity of this data was not significant (I^2^ = 0%; p = 0.49).”	“The heterogeneity of this data was not significant (I^2^ = 0%; p = 0.48).”
Results, Synthesis of results subsection, MIVC subheading, fourth sentence	“A small effect was seen between a-tDCS and Sham on MIVC (SMD = 0.29; CI_95%_ = 0.05 to 0.54; Z = 2.36; p = 0.02) with significant difference between the conditions.”	“A small effect was seen between a-tDCS and Sham on MIVC (SMD = 0.38; CI_95%_ = 0.10 to 0.65; Z = 2.68; p = 0.007) with significant difference between the conditions.”
Discussion, MIVC subsection, first paragraph, first sentence	“In our meta-analysis it was possible to demonstrate a small effect for MIVC between tDCS and Sham (ES = 0.29).”	“In our meta-analysis it was possible to demonstrate a small effect for MIVC between tDCS and Sham (ES = 0.38).”
Discussion, MIVC subsection, second paragraph, first sentence	“Previous studies using MIVC measures showed no difference between the a-tDCS and sham conditions [16, 17, 23, 26, 32–34, 42].”	“Previous studies using MIVC measures showed no difference between the a-tDCS and sham conditions [16, 17, 23, 26, 32–34].”

Additionally, the authors provide updated Figs 1 and 2 to address these errors. Please see the correct figures here.

**Fig 1:**
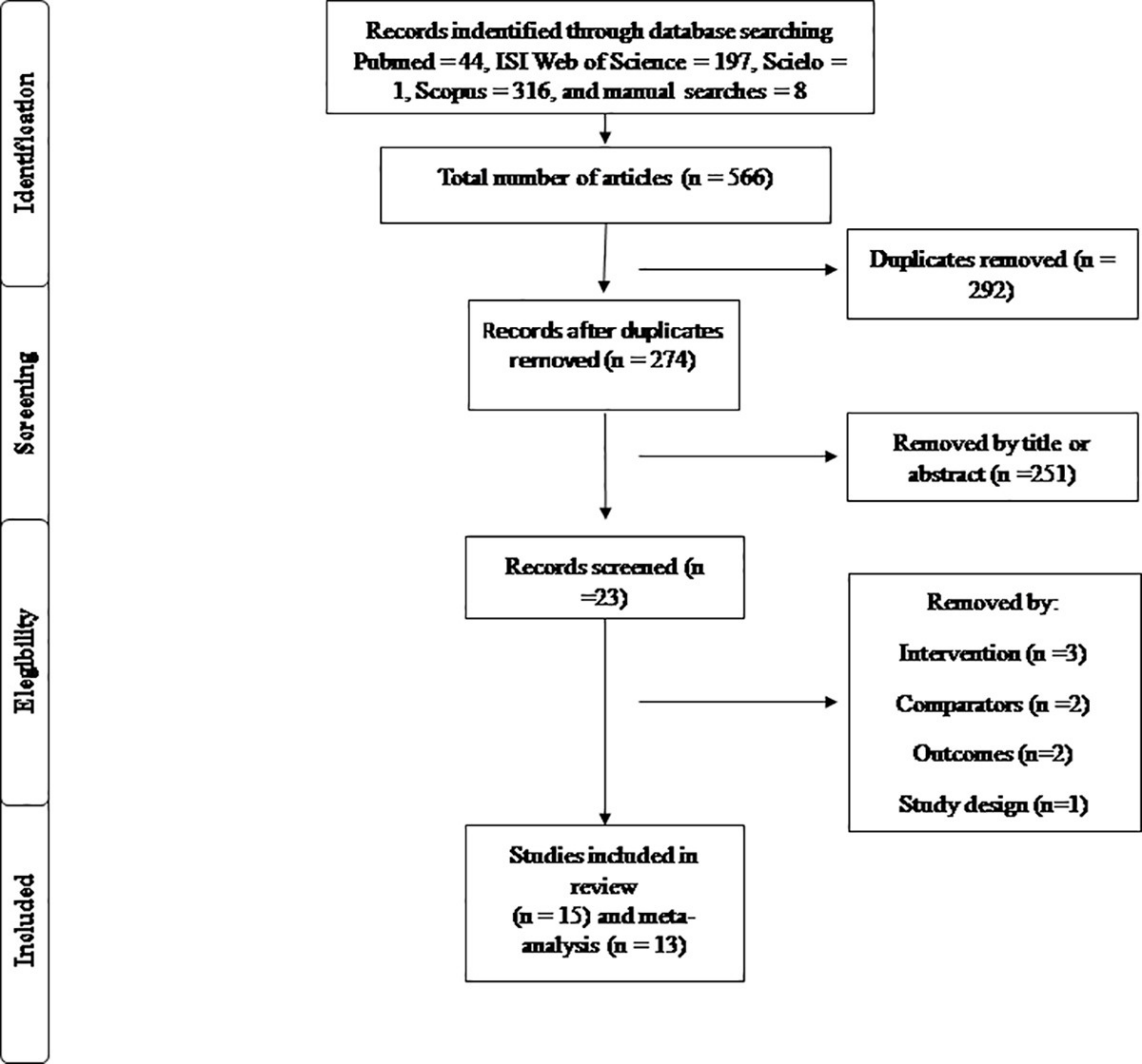
Flowchart of outcomes of search strategy.

**Fig 2:**
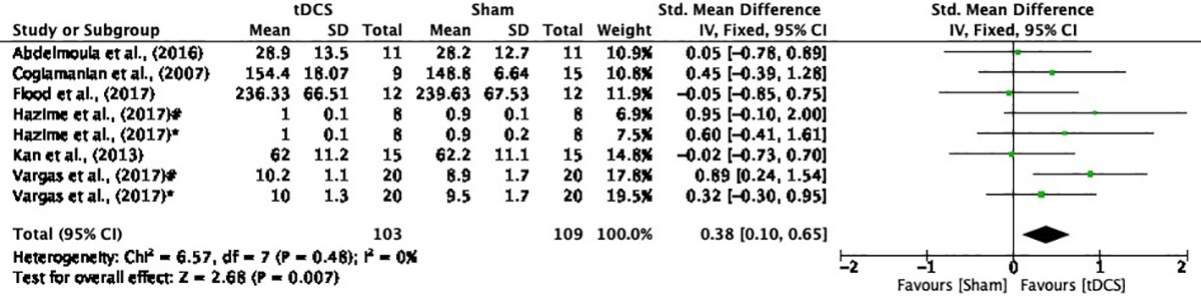
Forest plot showing a comparison of MIVC between tDCS and Sham. Hazime et al. (2017)#- internal rotador shoulder; Hazime et al. (2017)*- external rotador shoulder; Vargas et al. (2017)#- knee extensors dominant limb; Vargas et al. (2017)*- knee extensors non-dominant limb.

## References

[1] Lattari E, Oliveira BRR, Monteiro Júnior RS, Marques Neto SR, Oliveira AJ, Maranhão Neto GA, et al (2018) Acute effects of single dose transcranial direct current stimulation on muscle strength: A systematic review and meta-analysis. PLoS ONE 13(12): e0209513 10.1371/journal.pone.0209513 30586389 PMC6306262

